# Balancing Commercialization and Sustainability in Community-Based Tourism Practices - A Qualitative Study of Factors Affecting Elephant Habitat Communities in Northern Thailand

**DOI:** 10.3389/fpsyg.2021.685426

**Published:** 2021-07-06

**Authors:** Yu-Chih Lo, Pidpong Janta

**Affiliations:** ^1^Department of Leisure Industry Management, National Chin-Yi University of Technology, Taichung, Taiwan; ^2^Renewable Energy and Efficiency Research Team, National Energy Technology Center, National Science and Technology Development Agency, Pathum Thani, Thailand

**Keywords:** community-based tourism, wildlife, Northern Thailand, thematic analysis, qualitative study

## Abstract

Community-Based Tourism (CBT) offers local residents opportunities to manage local tourism resources while sustaining their lifestyle at the same time. The research objective of the study was to explore the process and experience of communities in Northern Thailand which are known as elephant habitats, how these communities strive for stimulating the local economy without jeopardizing the way of life. The study was qualitative in nature. Qualitative data collection methods such as field observations and in-depth interviews were employed. The qualitative data were further analyzed with thematic analysis. In practicing CBT, the findings identified positive factors (Establishment of Elephant Camps), negative factors (Waste from Tourism Activity and Effects of Global Crisis on Employment and Local Income), and suggestions (Waste and Environment Management). The study found that the communities took pride in their cultural as well as natural resources and they are willing to commercialize these resources to a certain degree, i.e. founding elephant themed facilities, as has evidently been indicated. Consequently, as many issues factor into the practice of CBT, the study concluded that community participation and government support should have played a crucial role in maintaining new balance of overall local lifestyle sustainability and commercialization during and after the pandemic.

## Introduction

The tourism industry has seen a change in tourist behavior in recent decades. Tourists look to explore destinations in a novel way in which they can absorb local culture and immerse themselves in their visits. A study found that when social needs are derived from human behaviors, tourism practices will receive increasing attention (Salazar and Cardoso, 2019). Having that in mind, in order to fulfill these social needs, tourists are willing to travel, not only to explore new or distant inexperienced destinations, but also looking for authentic experience with local people and communities (Sin and Minca, 2014). Thailand is a Southeast Asian country with unique culture and tradition, and researchers have found that tourists visiting Thailand tend to go beyond ordinary travel routes and tourist activities just to have meaningful as well as personal travel experiences (Lo and Janta, 2020).

### Growing Popularity in Wildlife Tourism

In Thai culture, elephants play an important role socioculturally. In terms of the tourism industry, elephants, undoubtedly, have become a tourist sensation for Thailand in recent decades. The rise of tourist interest in elephants has become a highlight in “wildlife tourism” in Thailand. According to a study (Kontogeorgopoulos, 2009b), wildlife tourism was defined as “tourism undertaken to view and/or encounter wildlife” and offers an unique interactive experience with animals in a natural habitat or in the wild. Despite the fact that certain degree of wilderness might cause inconvenience when enjoying the experience, the popularity and annual growth rate of wildlife tourism remain growing steadily among tourists (Dell'Eva et al., 2020). Several reasons contribute to the trend of wildlife tourism, such as accessibility of transportation, better communication technology, and friendly and supporting driving policies and services have made it easier for tourists to reach areas where wildlife is located (Kontogeorgopoulos, 2009b). On a social and psychological level, Kontogeorgopoulos (2009a) observed an interesting change in tourist behaviors and social attitudes toward nature and wildlife, i.e., as tourists have more interaction and experience with wildlife in natural settings, they are more likely to show a higher satisfaction toward the whole travel experience, then such experiences attract more tourists, and increase the time they tend to spend on their visit.

However, for wildlife tourism, the real challenge is to maintain tourists' satisfaction level and returning rate. To meet this challenge, a simulated natural facility, such as elephant nursery or camp presents a possible solution. In Thailand, Kontogeorgopoulos (2009a) pointed out that elephants in elephant nurseries or camps require a high quantity and quality of food e.g. 100 and 120 kg (220 to 440 pounds) of food per day. With the limited support and resources, in order to sustain the wildlife protection operation, elephants are trained to perform simple tasks to attract visitors. The selected elephants are tamed and trained in a harmless way and by mahouts (elephant babysitter) and fed with adequate nutrition (Kontogeorgopoulos, 2009a). After all, as much as elephant nurseries like to provide as authentic an interaction with the wildlife animals as possible, safety remains the top priority (Kontogeorgopoulos, 2009a).

However, the global pandemic of Coronavirus Disease 2019 (COVID-19) presented an even tougher crisis. The global tourism industry has been severely damaged by the pandemic. Specifically speaking for the elephant nursery, wildlife tourism in Thailand, suspension of flights, collapse of the hospitality industry, loss of tourists' traveling confidence, and lack of instant revenue, etc., all these have jeopardized the wildlife tourism business as well as the wildlife protection operation (Jones and Comfort, 2020). Elephant nurseries' or camps' owners have suffered tremendously in this pandemic crisis. With their business operation at risk and no or rather limited government financial support, elephant nurseries and the communities where they are located, with whom together they offer additional local cultural and natural resources, are all now facing the crisis of being self-sustaining.

Having this background in mind, the following sections explain further regarding the concept of community tourism as alternative means to tourism in developing countries, wildlife tourism, decline in elephant nurseries and camps, and new challenges brought by the global COVID19 pandemic. Moreover, the researchers give details about research methodology such as research design, data collection and methods, data analysis, and description of research sites. The study analyzed the qualitative data with thematic analysis and generated three themes from interviewees' responses: positive factors, negative factors, and suggestions. Finally, the discussion and conclusion explain how the findings factor into the tourist context and how they call for future studies.

## Literature Review

### Community-Based Tourism

Community tourism (sometimes called community-based tourism: CBT), is an alternative form of tourism aiming at sustaining tourism development and provides local residents with an opportunity to manage and control natural and cultural resources (Lo and Janta, 2020) in order to promote the local economy. Studies have suggested CBT as a sustainable model for maximizing local socio-economic benefits of tourism and minimizing negative environmental impacts (Ruiz-Ballesteros and Hernandez-Ramirez, 2010). In natural resource dependent developing countries, CBT also provides an opportunity for development in rural and remote areas by strengthening small individual business operations even without much government support (Tolkach and King, 2015). However, CBT does not lack weaknesses. Studies have suggested that practice of CBT might cause low profitability, inadequate contribution to community development, and weak marketing and promotion (Beaumont and Dredge, 2010).

The concept of CBT first appeared in Murphy's study and the same author had researched to a greater extent in 2004 (Yang, 2007). Basically, CBT practice tends to achieve four objectives: (1) Resources conservation: sustaining cultural and natural resources by bringing in positive impact to the community; (2) Socio-economic development: offering an opportunity for the community to balance costs and benefits; (3) Empowering community and its identity: strengthening community identity through meaningful activities that make residents own and recognize their local identity; and (4) High quality visiting experience: ensuring tourists receive authentic and meaningful experiences during their visit with environmental responsibility and awareness (Lo and Janta, 2020). For decades, studies have supported CBT practice for rural area development, local economy advancement, and overall community involvement and initiatives in tourism planning.

CBT practices cannot be successful without community participation, especially when it comes to conserving natural, cultural, and social resources, and any community-initiated tourism practices. It also relates to support from stakeholders and government authorities. A high level of community participation enables infrastructure development, ecological awareness, and actions for natural resources, which are essentials for local tourism development Tseng et al. (2019). and Rizzolo (2017) argued that wildlife animals, as part of the community's natural resources, have gained significance in local tourism practice. Given humans' disconnection with nature caused by urbanization and technology, the desire and demand to reconnect with nature and interact with wild animals has risen, hence the wildlife tourism (Rizzolo, 2017).

### Wildlife Tourism

Examining from the CBT perspective, wildlife tourism offers interaction opportunities for local communities and tourists, and allows tourists and wildlife animals (Orams, 2002) to share an experience in a safe setting ranging from captive settings (artificially constructed by humans), to semi-captive settings (featured containment of some freedom of movement), to wild areas in the natural environment (Kontogeorgopoulos, 2009b). The value of wildlife tourism therefore lies greatly in the nature of the interaction between human and wildlife animals found in those settings. In Thailand, for those related to elephant-themed tourism practices, semi-captive facilities such as elephant nurseries or camps have become the mainstream wildlife tourism setting in tourism industry (Kontogeorgopoulos, 2009a). Northern Thailand, in particular, is known to be the home of three quarters of the country's captive elephant population, and hence serves as the center of an emerging wildlife tourism as well as wildlife animal protection center (Kontogeorgopoulos, 2009a,b).

However, wildlife tourism is not simply about the joy of experiencing or observing wild animals in close distance, but more importantly, hoping to raise awareness to protect the wilderness, nature, and environment (Dell'Eva et al., 2020). Ideally, the desire to get away from cities and reconnect with people and nature allows wildlife tourism to “push” people away from their usual leisure routine, and simultaneously, the authenticity of local culture and nature “pull” people into the community (Mutanga et al., 2017). Wildlife tourism business operators and service providers are well aware of such natural competitive advantages and have been promoting local economy and building up destination identity based on it (Dell'Eva et al., 2020). However, wildlife tourism affects tourist satisfaction in different ways because satisfaction and opinion about the tour are shaped and influenced by the experience tourists have with wildlife animals in a setting (Bigne et al., 2005). High level of satisfaction encourages future re-visits, i.e., the more positive an experience tourists have during their visit, the more satisfied they are, and the more likely they are to return (Arabatzis and Grigoroudis, 2010).

### Decline in Elephant Nurseries Or Camps

Wildlife tourism under the CBT scope depends on its wildlife animals, yet in the past century, Asian elephants have faced a drastic decline in numbers. The World Wide Fund for Nature (WWF) estimates 25,600 to 32,750 Asian elephants are in the wild and over 15,000 are in captivity (Kontogeorgopoulos, 2009a). The decline of the elephant population can also be observed in Thailand. In just over a century, the number of elephants in Thailand has declined precipitously from approximately 100,000 in 1900 to 4,450 nowadays (Kontogeorgopoulos, 2009a). 1,000 or so of the 4,450 elephants in Thailand today are in the wild, exclusively in national parks and wildlife sanctuaries. ~3,450 are domesticated. Kontogeorgopoulos (2009a) pointed out that the ongoing decrease in the elephant population in Thailand indicates six issues, regulatory issues, serious injuries, insufficient nutrition, social environment, training, and lack of quality personnel, such as mahouts. In Thailand, on the local level, the laws regarding domesticated elephant treatments are often neglected and the government support is limited (Kontogeorgopoulos, 2009a). Furthermore, animal rights and welfare are not reinforced or regulated in ownership, so elephants simply become private property with no particular provisions for animal protection (Lair, 1997), even lack of care or abandonment. Other than common injuries such as cuts and bruises caused by trees or rocks often seen in careless settings, domesticated elephants also suffer injuries from tourist activities. To carry tourists along in the wild, a bench needs to be attached along the elephant's back. Grown elephants can carry a weight of a maximum of 300 kg (661 pounds), but it is recommended that the maximum weight that elephants should carry is 200 kg (441 pounds). For those unfortunate elephants that are assigned to carry a particularly heavy tourist, the task can be injurious and dangerous (Kontogeorgopoulos, 2009a). However, differing from Thailand, elephant camps in Botswana offer valuable and interactive experiences with trained elephants. Elephant safaris in Botswana promote “back to nature” experiences to visitors by divorcing them from the global dynamics. The produced “wilder tourism” has been considered as an income source that can be relied on (Duffy, 2014).

### Coronavirus Disease 2019 (COVID-19) Crisis

Along with the potential crisis mentioned previously, COVID-19, the global pandemic the world is facing now, has posed an even greater obstacle for every industry sector. The common symptoms of the disease are fever, cough, shortness of breath, and general fatigue (Jones and Comfort, 2020). To manage the spread of the disease, public policy and legislation are designed and restrictions, namely locking down cities or borders, are common everywhere. As a result, loss of the necessities that support the whole tourism industry, makes it clearly difficult for elephant nursery owners or safari service operators to manage and to survive in the crisis of the pandemic (Ghaderi et al., 2012). Other than those who chose to close their business practices temporarily, to make the matter worse, the permanent loss of employment in the tourism hospitality related industry is estimated at 48.7 million in the Asia Pacific region, especially in countries relying heavily in tourism, such as Thailand (Jones and Comfort, 2020). Before adapting to the new normal forced by the pandemic crisis, the domino closing effect of tourism and hospitality industry related business, sadly, still continues (Baum and Hai, 2020).

### The Importance of Place

Practicing CBT along with wildlife tourism emphasizes the significance of geographic entities i.e. space and place (Tapsell and Tunstall, 2008). Place indicates a socially constructed idea, scripted with certain rules of accepted behavior (Crang, 2014). The rules are expected to be followed so an individual can find the senses of belonging and dynamic offered by the place (McCabe and Stokoe, 2004). The value of place depends on the interaction between space and individuals or social groups in it (Deery et al., 2012). Tourism serves as a platform for place change, thus creating emotional ties between tourist and destination (McKercher et al., 2015). To successfully practice CBT and wildlife tourism, it is important to acknowledge the importance of place identity, place dependence, and place attachment (Jorgensen and Stedman, 2001).

#### Place Identity

While an individual's identity is formed through “a complex pattern of conscious and unconscious ideas, beliefs, preferences, feelings, values, goals and behavioral tendencies” (Proshansky, 1978), four features of place identity were identified (Twigger-Ross and Uzzell, 1996) including (1) distinctiveness: summary of continuity of self and feeling that a person has a place-referent relationship with environment; (2) continuity: defined as a reflection of an individual's desire to retain a connection over time which can foster psychological well-being; (3) self-esteem: feelings of worthiness or social value; and (4) self-efficacy: a personal desire to remain in the environment that facilitates or at least does not hinder a person's lifestyle. Having this in mind, a good tourism destination identity serves as an agent to fulfill an individual's search of identity (Twigger-Ross and Uzzell, 1996).

#### Place Dependence

Place dependence was developed and recognized as individual perceived strength associated with him or herself within specific places (Stokols and Shumaker, 1981). Place plays a critical and functional role to serve an individual. Individuals usually develop stronger place dependency when the place suits their needs (Stokols and Shumaker, 1981). A successful practice of CBT and wildlife tourism creates strong place dependence by meeting the tourists' desire to reconnect to nature and search for authentic local cultural immersion (Arabatzis and Grigoroudis, 2010).

#### Place Attachment

The final element of place, place attachment, reflects a positive bond or relationship between groups of people or among individuals within a social environment (Low and Altman, 1992). Place attachment can also be interpreted when an individual finds a sense of belonging (Gustafson, 2006). Moreover, attaching to a place or not could also depend on the degree of insidedness and outsideness (McKercher et al., 2015). In short, the more an individual feels like an insider, the more he or she is likely to feel a strong sense of fondness or connection in a place, and vice versa (McCabe and Stokoe, 2004).

In a tourism context, a place or a tourism destination is constructed on the basis of tourist experience, place, and other socio-economic connotations (McKercher et al., 2015). Tourists tend to look for a sense of insidedness where they are welcome, and fondeness to where or what they can attach. Hence, changes in a place can affect individuals' perceptions and attitudes toward a place directly. To benefit CBT and wildlife tourism practices, it is crucial to first enhance overall quality of life of a place or a community, to deepen residents' place attachment and make positive place change so to speak. On the other hand, negative impact is associated with decrease in place attachment, lost of distinctiveness, self-esteem or self-efficacy (McKercher et al., 2015). In summary, a negative relationship between individuals and negative changes in places can lead to loss of distinctiveness and damage to value of a place, resulting in being less desirable by outsiders (Gilbert and Clark, 1997) and the discontinuation of local culture, and a successful CBT and wildlife tourism practice needs them all.

## Methodology

### Research Design and Methodology

**Figure 1:**
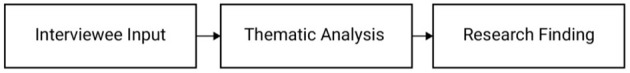
Research methodology employed in study.

Figure 1 illustrates research methodology in the present study. A qualitative research design was employed, relying on interviews and thematic analysis (Braun and Clarke, 2006). As the value of qualitative research is increasingly being recognized, qualitative methodology intends to generate knowledge based on human experiences and offer insights which suitably respond to the question of why people engage in certain actions. This kind of methodology is suited to analyze assumptions from qualitative data sets and obtain meaningful and useful results.

Data collection through a semi-structured interview was administered because the study aimed to understand how community-based tourism practices are affected and how groups of people interact and behave in a certain action. Interviews were conducted in the communities where the elephant nurseries and camps are located. The study focused on interviewing local business owners whose business practices are directly or indirectly affected by the rise and fall of the elephant nurseries and camps. The majority of the previous studies relating to this research purpose were all based on quantitative methodology, thus, aiming at contributing a different view point, the qualitative method was employed in the study. Additionally, face-to-face interviews allowed interviewees to clearly understand the questions and the purpose of this research. Unexpected and uncalculated responses are all welcome. The interviewees were invited to speak as they please.

With the purpose of identifying factors that impact the commercialization and sustainability of CBT practices in wildlife tourism areas, in this case elephant nurseries or camps, the research objectives of the study are as follows:

- Exploring factors affecting the CBT practice in wildlife tourism areas considering the global pandemic.
- Conceptualizing the factors and their effect on commercialization and sustainability in order to further CBT development in the wildlife tourism area with the concern of the global pandemic.

Based on literature on wildlife tourism, many attempts were made to understand how business is affected by the inexistence and existence of elephant camps in a specific area. However, most of the studies have not yet explored the issues considering the global pandemic crisis.

### Data Collection

As seen in Table 1, the characteristics of interviewees were sorted into Occupation and Year of Work Experience. Semi-structured face-to-face interviews were conducted with business owners, employees, managers of elephant camps, and community members. The duration of the interviews lasted between thirty to fifty min. Consent from the interviewees was obtained and note-taking and videotaping and transcriptions were also permitted.

**Table 1:** Interviewee information, occupation and year of work experience.

**No**.	**Sources**	**Occupation**	**Year of work experience**
1	Interviewee A	Business owner	5–10 years
2	Interviewee B	Employee	10–15 years
3	Interviewee C	Retailer	5–10 years
4	Interviewee D	Retailer	5–10 years
5	Interviewee E	Retailer/business owner	Less than 5 years
6	Interviewee F	Business owner	10–15 years
7	Interviewee G	Employee	Less than 5 years
8	Interviewee H	Business owner	More than 15 years
9	Interviewee I	Retailer/business owner	10–15 years
10	Interviewee J	Business owner	Less than 5 years
11	Interviewee K	Retailer	5–10 years
12	Interviewee L	Junior manager	More than 15 years
13	Interviewee M	Employee	More than 15 years
14	Interviewee N	Retailer	5–10 years
15	Interviewee O	Business owner	5–10 years
16	Interviewee P	Officer (elephant camp)	5–10 years

Business owners were invited to share their opinions freely regarding the impacts that elephant nurseries or camps have made in the nearby communities. In order to understand interviewees' responses better, the interviewees were provided with the guided interview questions in writing as well. The researchers prepared guided interview questions such as: what are the factors affecting community-based practices in the perspective of elephant habitants? Additionally, questions regarding Pre- and Post- Elephant Establishment, Tourism Behavior, Pre- and Post- Coronavirus Outbreak, The Second Wave of Coronavirus Infection, Other Related Pandemics, and Suggestions, were given too. The study, qualitatively designed in nature, with a face-to-face semi-structured interviewing technique, hoped to contribute to a different and deeper understanding of CBT practices in elephant habitats with the current pandemic crisis in mind.

### Data Analysis

The study analyzed the collected qualitative data and explored patterns with Braun and Clarke's thematic analysis approach (Braun and Clarke, 2006). The Braun and Clarke thematic analysis suggested six phrases: revisiting collected data, generating codes, establishing themes, re-examining themes, defining themes, scripting, and reporting findings. Furthermore, in addition to the thematic analysis, considering the weakness of qualitative research, the criteria for trustworthiness developed by Lincoln and Guba were applied in each thematic analysis phase (Lincoln and Guba, 1986) to strengthen the validity of the study.

### Research Area

In Thailand, there are ~40 to 50 elephant nurseries/camps/parks (Tipprasert, 2002), however the COVID-19 pandemic has forced most of them out of operation. In Chiang Mai Province in Northern Thailand, Seven elephant camps (with more than 15 elephants) are recognized as major camps in these three districts: Mae Rim, Mae Taeng, and Chiang Dao (Kontogeorgopoulos, 2009a). The study started the data collection from September 2020 to March 2021. The city lockdowns, travel restrictions, and quarantine policy during the time certainly made the data collection less than easy. The researchers focused on these two communities: Mae Taman Community and Kuet Chang Community in Chiang Mai Province. The targeted communities are several nearby wildlife tourism business operations and elephant centers, such as Mae Taeng Elephant Park and Maesa Elephant Camp. In addition, the two communities also lie within close proximity (about 2-h drive) to the major city of the province. Therefore, they are the ideal research sites for the study.

First, Maesa Elephant Camp has been around since 1976, first as a conservation center for domesticated elephants rescued all over Thailand. Eventually, the camp expanded to include Maesa Elephant Nursery and Thai Elephant Care Center. At present, there are 78 elephants under the care of the facility. The founding purpose of the center is to make the land an elephant's land again. Due to the COVID-19 pandemic, the camp owner decided to stop all the elephant related tourist activities.

Next, Mae Taeng Elephant Park was established in 1989. Back at the time, the Thai government reinforced a nationwide logging ban, which resulting in work elephants in the logging industry roaming the area abandoned. The Mae Taeng Elephant Park founder and the family decided to create a safe haven for the abandoned elephants. Another founding purpose of the park is to educate people about elephants, so visitors from all over the world can visit Mae Taeng Elephant Park and learn about the animals. Then in 2006, the park founded an elephant clinic specializing in elephant pregnancy. The park also collaborates with Lam Pang Elephant Hospital to support a fully equipped licensed clinic with a full time veterinary staff and contributing to animal science. With care and respect for elephants, the park has become a renowned wildlife tourism facility in Thailand.

## Research Finding

To explore the understanding of participants, thematic analysis was applied to analyze all the interviewee transcripts and themes were derived from the process. Based on research objectives, the themes were categorized as “Positive Factor,” “Negative Factor,” and “Suggestion.”

### Positive Factor

#### Establishment of Elephant Camps

Tourism products and attractions attract tourists. Places with a variety of attractions to offer tend to satisfy tourists, in this case communities that offer local culture, tradition, and wildlife definitely meet the needs of tourists (Bigne et al., 2005). As an interviewee stated “feeding elephants, natural atmosphere, and professional photographing service, and rafting down the river are popular activities that tourists love.” When a tourist attraction is well developed in a way that encourages tourists to spend more time and stay longer, it improves the local economy too. Another interviewee informed “before, our business was struggling because we didn't even have the necessary infrastructure like road with asphalt. Now, everything changed since the elephant camp opened. You can see business like strawberry farms, cafés in the waterfalls area or restaurants along the road.” From the analysis, interviewees vividly shared and explained the differences in life before and after elephant camps. Several interviewees all shared that “the major differences are such as better income, job opportunities, increase of different business and attractions in the elephant camp area.” Two interviewees who are business owners both reported that “they started their business after they realized the increasing flow of tourists visiting the camps or other destinations in the area. Local residents also took the opportunities to start business, make money, make a living, and find any way to improve their lives.” Another interviewee agreed and said “tourism attractions not only bring in money, transportation, and infrastructure, but also gave me a job opportunity. I work in the elephant camp souvenir shop, and the money I got from the job is enough to support me and my family.”

### Negative Factor

#### Waste From Tourism Activity

The tourism industry, in addition to serving its leisure and recreational purpose, sadly, also creates waste. “As we have more business operations for the tourists flow, the waste generated could be twice as much as we used to have,” an interviewee observed. Waste from the hospitality business and households compose of a mix of recyclable, non-recyclable, non-biodegradable, organic, and even hazardous materials that can harm the environment (McKercher et al., 2015). The interviewees pointed out that after elephant camps open for services, local residents have demanded that the park should be responsible for waste from all the tourist activities. An interviewee stated “with more and more tourists coming, with our own garbage, our trash load is more than our waste management can handle.” Another one further added “getting a lot of tourists does bring in a lot of money; however, it also brings in a lot of trash from the outside to us. What happens is, tourists left, the trash stays. So, part of the money we make has to spend on taking care of the waste. We become in charge of waste disposal and it costs too much sometimes. The trash situation got worse, as the elephant camp has attracted tourists all year round, there is no high and low season. The trash just keeps coming.” The interviewee further indicated that hospitality related business is a major contributor to the plastic and metal waste. “We provided food services and we take care of our own garbage. But, not every food place thinks like that. What happens is you see tourists just throw the trash on the street when they left. This is really disturbing and it's not good for the environment,” an interviewee added. According to the interviewees, to overcome the problem, different areas have implemented strategies based on their specific priorities in order to promote sustainability and increase efficiency of waste management systems. Studies have called for attention and urged taking actions to waste management for a sustainable environment (Rizzolo, 2017).

#### Impact of The Global Pandemic Crisis on Employment and Local Income

The impact of the COVID-19 crisis in Southeast Asia is unprecedentedly serious. Even now, policymakers and tourism practitioners are still helpless in reacting to the crisis. It is widely agreed that the impact of the COVID-19 pandemic on the travel and tourism industry is so severe that it is incomparable to the previous global crisis (Baum and Hai, 2020). Based on the pandemic situation, the economy worldwide will continue to slide. Loss of jobs and tourists has struck the local economy hard. All the interviewees agreed that the crisis has severely damaged all sectors of the local economy, and the pandemic acutely damaged the local tourism industry. An interviewee said “since the flights were banned and places were restricted, there were no tourists on street. My income was cut at least by half, I think it is fair to say, the COVID-19 pandemic killed the business.” Another interviewee reproached the government's actions and added “we lost our regular customers since the pandemic broke out. We cannot rely on someone, especially government authorities, to help us. It usually take some time before the help reach us. What we can do now is just to minimize the impact by isolating ourselves from another and keep ourselves healthy.” Furthermore, the government's quarantine and self-health management policies have caused lockdown across the country. Another interviewee reported “checkpoints were set up between communities. In order to move to another community, it is mandatory to apply for official permission from the local administrative agencies. Basically, we can't go anywhere.” The same interviewee continued “during that time, some businesses relied on online services. But, most local people are not familiar with getting things online. Most of employees were forced to let go so the owner can at least keep the business afloat at minimum cost.” These insights from the interviewees, again, showcased the severity of COVID-19 effects on the travel and tourism industry as well as the local community. Finally, an interviewee sadly mentioned about the elephants, “elephants lost all the care and welfare too because mahouts were all laid off and not so much budget available for the food and the training. There used to one mahouts taking care of two to three elephants, now, with most of them lost their jobs, I can't imagine what is going to happen to those elephants.”

### Suggestions

The research findings of this study presented that both positive and negative factors can have an impact on the commercialization and sustainability of CBT practices in the studied wildlife animal areas. Essentially, this study suggested that waste management issues needed to be addressed in order to promote sustainability. Based on the findings, solutions were suggested by the interviewees.

#### Waste and Environment Management

Tourism is the pillar industry in northern Thailand. Waste management holds a hidden critical role in providing a better tourist experience. A clean community naturally attracts tourists. In the study, over half of the interviewees reported that waste derived from tourist activities is the major concern that calls for immediate action. Three out of 16 interviewees suggested improving waste disposal and systems. One stated “it is necessary to manage waste in a proper way. Since the local residents have all tried to reduce the amount of waste they produced, so improving waste processing infrastructure can be an action for now to preserve the beauty and health of our place.” Whereas, another interviewee said “there should be implementation of a national waste management policy to control and limit the amount of waste produced by tourism providers.” By the same token, the study suggested that there is need to set up waste sorting and collection facilities at the local level as well as waste management policies.

## Discussion and Conclusion

The study concluded that wildlife tourism, as in elephant nurseries or camps serves as a beneficial factor to the nearby communities' CBT practices. Establishment or promotion of a wildlife destination brings in large amounts of income, jobs, and business opportunities to local economy. Additionally, it also allows the elephant caring facilities to sustain themselves (Kontogeorgopoulos, 2009a). However, despite the benefits, the study found that such commercialization reveals inadequate public infrastructure, inaccessibility to the destinations, services capacity, insufficient human resources, lack of community participation, and most importantly, waste management.

On the other hand, in addition to the waste management issue, the study found a negative factor inflicting depressive effects on the tourism sector and the quality of tourist destinations. Undeniably, the global pandemic crisis has impacted tourism destinations and local residents' lifestyles. Both residents in the communities and tourists from outside the communities are restricted to prevent the spread of the disease. Lockdowns have taken away any tourist related opportunities for the local communities. However, the pandemic will end, for tourism business operators to manage a crisis like the COVID-19 pandemic it is important to empower managerial personnel. To keep the business operation running for the time being, it is crucial to manage the risk, devise a contingency plan, damage control, and a proactive recovery plan, (Ghaderi et al., 2012). Lack of governmental immediate support and effective actions, close-down of tourist destinations and economic depression are expected and the damage to the overall tourism industry is long term. Therefore, to recover from the pandemic, the community may adopt marketing initiatives, media policy, and advertising campaigns to restore a positive image (Avraham, 2015). Alternatively, it needs a strong collaboration from the government, private sector, even international organization, together, to boost the economic growth (Duffy, 2014) and start a new normal and new balance in lifestyle.

## Data Availability Statement

The raw data supporting the conclusions of this article will be made available by the authors, without undue reservation.

## Ethics Statement

Ethical review and approval was not required for the study on human participants in accordance with the local legislation and institutional requirements. The patients/participants provided their written informed consent to participate in this study.

## Author Contributions

Y-CL made substantial contributions in exploring research concept, developing the research framework, and designing the methodology. PJ made substantial contributions in data collection, writing the manuscript, and searching for references. All authors made substantial contributions in data analysis, result interpreting and discussing, drafting the manuscript, and writing the authors' response notes. All authors contributed to the article and approved the submitted version.

## Conflict of Interest

The authors declare that the research was conducted in the absence of any commercial or financial relationships that could be construed as a potential conflict of interest.
